# Improved assay to detect *Plasmodium falciparum* using an uninterrupted, semi-nested PCR and quantitative lateral flow analysis

**DOI:** 10.1186/1475-2875-12-74

**Published:** 2013-02-22

**Authors:** Serge Y Ongagna-Yhombi, Paul Corstjens, Eran Geva, William R Abrams, Cheryl A Barber, Daniel Malamud, Sungano Mharakurwa

**Affiliations:** 1 Department of Basic Science, NYU College of Dentistry, New York, NY, 10010, USA; 2 Department of Molecular Cell Biology, Leiden University Medical Center, Leiden, The Netherlands; 3 Johns Hopkins University, Bloomberg School of Public Health, W Harry Feinstone Department of Molecular Microbiology and Immunology, Baltimore, USA; 4 The Malaria Institute at Macha, PO Box 630166, Choma, Zambia; 5 Current address: University of Delaware, Department of Biological Sciences, Newark, DE, 19716, USA

**Keywords:** Malaria, Detection, Semi-nested PCR, PCR, Lateral flow, *Plasmodium falciparum*

## Abstract

**Background:**

A rapid, non-invasive, and inexpensive point-of-care (POC) diagnostic for malaria followed by therapeutic intervention would improve the ability to control infection in endemic areas.

**Methods:**

A semi-nested PCR amplification protocol is described for quantitative detection of *Plasmodium falciparum* and is compared to a traditional nested PCR. The approach uses primers that target the *P. falciparum* dihydrofolate reductase gene.

**Results:**

This study demonstrates that it is possible to perform an uninterrupted, asymmetric, semi-nested PCR assay with reduced assay time to detect *P. falciparum* without compromising the sensitivity and specificity of the assay using saliva as a testing matrix.

**Conclusions:**

The development of this PCR allows nucleic acid amplification without the need to transfer amplicon from the first PCR step to a second reaction tube with nested primers, thus reducing both the chance of contamination and the time for analysis to < two hours. Analysis of the PCR amplicon yield was adapted to lateral flow detection using the quantitative up-converting phosphor (UCP) reporter technology. This approach provides a basis for migration of the assay to a POC microfluidic format. In addition the assay was successfully evaluated with oral samples. Oral fluid collection provides a simple non-invasive method to collect clinical samples.

## Background

Malaria, a mosquito-borne disease caused by parasites of the *Plasmodium* genus, exacts a global toll of at least 216 million clinical cases and 655,000 deaths annually of which ~85% are children under the age of five [1]. Currently, diagnosis of malaria is based on four different approaches: microscopy, antigen detection using immuno-chromatographic rapid diagnostic tests (RDTs), malaria antibody detection, and nucleic acid-based assays. Microscopy remains the “gold standard” method for laboratory confirmation of malaria, and involves examination of thick and thin blood films stained with Romanowsky stain (mainly Giemsa or Field stain). However, microscopic diagnosis of malaria is dependent on the personnel performing and interpreting the results, and requires specialized equipment that is difficult to support in remote areas lacking a reference laboratory. Moreover, low parasitaemia, especially in asymptomatic subjects, is not detected by microscopy.

To alleviate some of the difficulties of microscopy-based diagnosis of malaria, RDTs that detect parasite-specific antigens were developed [2]. The most commonly targeted malaria antigens are *Plasmodium falciparum* histidine-rich protein-2 (pfHRP2) and *Plasmodium* lactate dehydrogenase (pLDH) [3-6]. RDTs offer ease of operation, a timely diagnosis, and do not require trained personnel or special equipment [2,7]. However, they are relatively expensive and prone to false-positive responses due to persistence of pfHRP2 antigen in blood for up to two weeks after the parasite is cleared [2,8]. Also, the relatively low RDT sensitivity is a constraint for endemic regions attempting malaria pre-elimination, where detection and treatment of low-grade reservoir infections is required for effective elimination of infection [9].

In recent years, a molecular approach has been used to detect *Plasmodium* nucleic acids circulating in blood, saliva, and other body fluids [10-13]. Polymerase chain reaction (PCR) is more accurate and sensitive than microscopy and RDTs, detects low-grade parasitaemia and is indicative of active infection [14,15]. Detection and amplification of *Plasmodium* DNA is generally performed using nested PCR, a two-step procedure in which the product of the initial reaction is amplified a second time with a new pair of “inner” primers that hybridize to the dihydrofolate reductase (DHFR) gene located within the previously amplified region [10,16]. Nested PCR typically requires transfer of a small amount of product from the first step to serve as template for the second amplification in a second tube. The requirement to transfer PCR-amplified products dramatically increases the risk of carry-over and environmental contamination. Moreover, the two rounds of amplification may require up to six hours to complete. Investigators have attempted to develop single-tube nested or closed-tube nested PCRs to eliminate the transfer procedure and thus minimize contamination, reducing false-positive results, and maintaining high sensitivity [17]. However, to date, despite the high sensitivity and low risk of carry-over contamination associated with a single-tube nested PCR, this technique is susceptible to inhibition by inappropriate sample preparation [17]. Real-time PCR also minimizes contamination, but nesting is needed to optimize the limit of detection (LOD) [18,19]. Also, the common use of SYBR Green DNA-intercalating dye in many real-time PCR kits, which binds any double strand DNA, makes the assay less specific and prone to false-positive results. Furthermore, the use of specific fluorescent probes for certain types of qPCR, although specific, are expensive which greatly diminishes their routine use [20].

A generic platform [21-23] was developed to analyse HIV and other pathogen-related antigens, antibody, and nucleic acids in saliva or blood simultaneously by a combination of RT-PCR and an immunoassay with detection by up-converting phosphors (UCP) label and lateral flow technology [21,24-26]. The UCP reporter converts photons of lower energy infrared light into higher energy visible light and is ultrasensitive since this unique process does not demonstrate autofluoresence [27]. Using a similar approach an uninterrupted, asymmetric, semi-nested PCR providing quantitative detection of a *P. falciparum* DNA target with a significant reduction in overall assay time and improved robustness with respect to reducing the probability of contamination was developed. The system is ideal for further development to a point-of-care (POC) device.

## Methods

### Samples and instrumentation

DNA from *P. falciparum* laboratory clone 3D7 was provided by Professor Karen Day, New York University School of Medicine. The 3D7 DNA was adjusted to a working concentration of 270 ng/μl and used as positive control for the PCR assays. Unstimulated whole saliva samples (5 ml) were collected in the area surrounding the Malaria Institute at Macha (MIAM, Choma, Zambia) from patients tested positive for malaria by microscopy [11]. The specimens were transported to the Malaria Institute at Macha laboratory within a few hours of collection, where they were aliquoted into 1 ml amounts and stored at −20°C. Ten-fold serial dilutions of *P. falciparum* NF54 culture, donated by Dr Godfree Mlambo, Johns Hopkins Bloomberg School of Public Health, were used to determine the parasite detection threshold, and negative controls lacking template were included to preclude contamination. All amplifications were performed using a DNA engine DYAD PCR (BioRad). Digital quantification of amplicon from gel images employed ImageJ [28] or ImageQuant software (ImageQuant, GE Healthcare Life Sciences, Piscataway, NJ, USA).

### Isolation of malaria DNA from saliva and dried blood

Whole saliva samples (1 ml) were centrifuged at 8,000 rpm for 3 min. Essentially all of the DNA was in the pellet, which was then extracted using the crude cell lysate protocol of the Qiagen DNAeasy® Blood and Tissue kit (Qiagen, Valencia, CA, USA) according to the manufacturer’s instructions and DNA eluted in 8 μl of distilled H_2_O.

Whole blood samples were spiked with a titer of *P. falciparum* parasite and then ~100 μl was dried on Whatman filter paper. A Qiagen QIAamp DNA mini kit was used to isolate parasite DNA from six 3-mm diameter punches (Harris Punch) from the dried filter paper in a total volume of 150 μl distilled H_2_O.

### Primers used for PCR amplification reactions

The initial studies employed genus-specific primers rPLU1/rPLU5 for the small ribosomal subunit that yields a 1.6 kb fragment for first amplification (rPLU1: 5^′^-TCAAAGATTAAGCCATGCAAGTGA-3^′^ and rPLU5: 5^′^-CCTGTTGTTGCCTTAAACTCC-3^′^). The inner species-specific labelled primers for the second amplification yielded a 205 bp fragment for *P. falciparum* were rFAL1 (Forward): 5^′^-Digoxigenin-TTAAACTGGTTTGGGAAAACCAAATATATT-3^′^ and rFAL2 (Reverse): 5^′^- Biotin-ACACAATGAACTCAATCATGACTACCCGTC-3 [10,16,29-31]. With these primers the LOD was only 1.35 ng of DNA. Subsequent experiments used a second set of *P. falciparum* DHFR gene specific primers: U1 (Forward: 5^′^ -GGAAATAAAGGAGTATTACCATG-3^′^) and U2 (Reverse: 5^′^-Biotin-TAAGGTTCTAGACAATATAACA-3^′^), which yields a 273 bp fragment in the primary amplification. Inner primer U3 (Forward: 5^′^-Digoxigenin-GAAATGTATTTCCCTAGATATGgAATATT-3^′^) and U4 (Reverse: 5^′^-Biotin-ATTTATCCTATTGCTTAAAGGT-3^′^) [10,16,30-34] were used in the second round of amplification and produced a 229 bp amplicon. For the semi-nested asymmetrical PCR the U1 primer was labeled with digoxigenin. The PfDHFR primers were adopted as they attained superior detection threshold in parasite serial dilutions and in earlier work detecting *P. falciparum* from human saliva samples [11]. The primers also flank DHFR amino acid codon 108 for optional detection of anti-folate drug resistance-associated mutations.

### Molecular detection of *Plasmodium falciparum*

**Figure 1:**
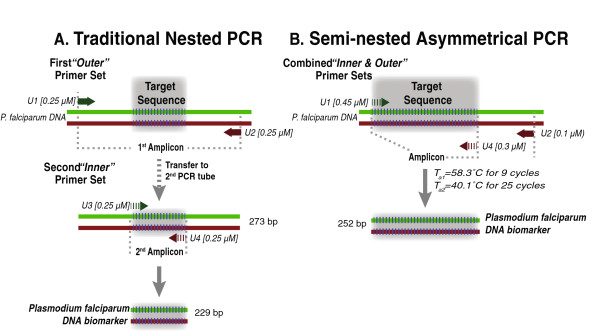
**Schematic of nested PCR (A) and semi-nested asymmetric PCR (B).** Note that the semi-nested PCR does not require a transfer of the first amplicon product.

A gene-specific nested PCR protocol developed by Dr. David Sullivan’s group [11] was used in a conventional nested PCR as illustrated in Figure 1. To allow detection of *P. falciparum* PCR amplicons by lateral flow, forward primer U3 and reverse primer U4 were synthesized with biotin and digoxigenin haptens at their respective 5^′^-ends. The resulting biotin-digoxigenin tagged DNA amplicon was bound to UCP reporter particles coated with mouse-anti digoxigenin [35] and subsequently captured by an avidin test line on nitrocellulose lateral flow strips. The UCP fluorescence signal was detected by interrogating the strips with a 980 nm laser [35]. The ratio of the test line to the control line UCP signals observed is proportional to the amount of target molecule.

### Nested PCR amplification

Conventional nested PCR for *P. falciparum* with the primer pair U1 and U2 yielding a 273 bp amplicon (Figure 1A) was carried out as described by Mharakurwa *et al.*[11]. Using the 273 bp amplicon as a template, the second round of PCR employed the nested primer pair U3 and U4 that amplifies a nested product specific for *P. falciparum*.

Briefly, both the first and second PCR amplifications were carried out in a total volume of 25 μl consisting of 2 μl template (0.5 μl for standard lab clone 3D7); 0.25 μM of each primer; 1.5 mM magnesium chloride; 0.2 mM dNTPs; 1X PCR Buffer; and 1U Taq polymerase. The nested PCR amplification required a total of six hours to complete both the first and second amplifications. Using conventional nested PCR (Figure 1A), an initial amplification was performed using primers U1 and U2 and the following programme: 94°C/2 min; (94°C/45 sec; 43°C/45 sec; 65°C/1 min) × 25 cycles after which 1.6 μl was transferred to a second tube with fresh reagents and inner primers U3 and U4. Amplification using the same program was performed for an additional 25 cycles for a total run time of ~3.5 hours.

### Semi-nested asymmetric PCR

Unlike conventional nested PCR, the semi-nested asymmetric PCR (Figure 1B) was performed in a single tube, uninterrupted without transfer of amplicon using the following programme: 94°C/2 min; (94°C/45 sec; 58°C/50 sec; 65°C/1 min) x 9 cycles; 94°C/2 min; 94°C/45 sec; 40°C/50 sec; 65°C/1 min) x 25 cycles; 65°C, 2 min with a total running time of ~1.5 hours. The single-tube semi-nested PCR reaction, which generates a 252 bp amplicon, was performed in a total volume of 20 μl containing 8 μl template DNA; 0.45 μM, 0.1 μM and 0.3 μM, respectively of primers U1, U2 and U4; 1X PCR buffer, 0.2 mM dNTP; 2U Taq polymerase.

### Lateral flow assay adaptation

The PCR-generated digoxigenin-biotin labelled amplicon was analysed as previously described [35]. Briefly, 1 μl of amplified PCR product was mixed with 100 ng mouse-anti digoxigenin-UCP conjugate dispersed in 100 μl of lateral flow buffer (100 mM Hepes pH 7.2, 270 mM NaCl, 1% (w/v) BSA, 0.5% (v/v) Tween 20) and incubated in a thermal shaker (1,000 rpm, 37°C) for 30 min. The mixture was then added to a microtiter plate well containing a lateral flow nitrocellulose strip with a “test” line consisting of avidin and a “control” line of anti-mouse-digoxigenin [35]. The avidin test line captures the biotin labelled PCR amplicons bound to the UCP reporter as it flows on the lateral flow strip and the unbound UCP binds to the distal control line comprised of mouse anti-digoxigenin antibody. Following immunochromatography, the strips were dried and analysed for UCP using a Packard Fluoro Count™ adapted with a 980 nm Infrared Opto Power Corp laser [27].

## Results

**Figure 2:**
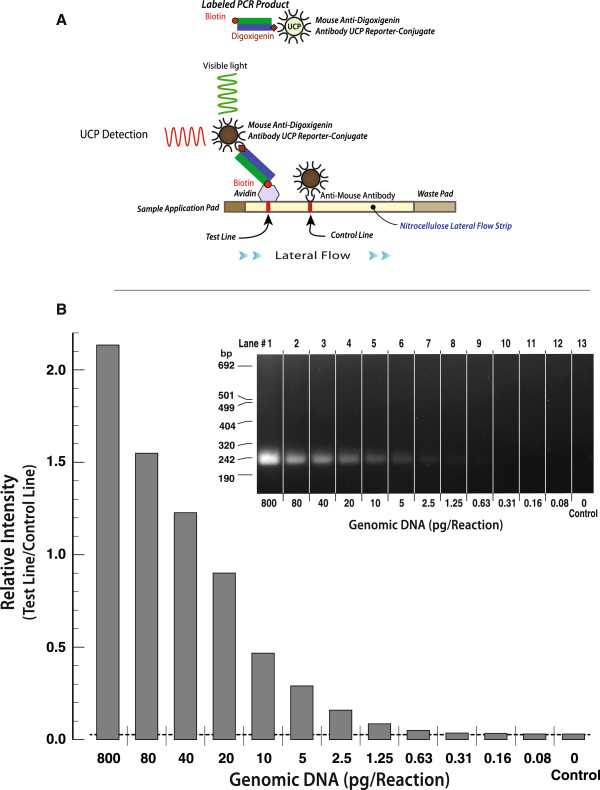
**PCR titration of genomic DNA using the genus specific primers. A**) The schematic outlines the strategy for UCP detection. **B**) UCP detection of digoxigenin-biotin labelled amplicon generated from a serial dilution of *P. falciparum* DNA using semi-nested PCR. The equivalent # parasites per reaction were 38084, 3408, 1704, 852, 426, 213, 107, 53, 27, 13, 7, and 3, which corresponds to Lane numbers 1 through 12 on the image of the agarose gel. The insert is the ethidium bromide stained agarose gel (2%), which confirms the resulting amplicon products as a function of the serial dilution of *Plasmodium falciparum* DNA. Each lane contained 18 μl of the PCR product. The intensity of the phosphorescent UCP reporter particles was measured through excitation at 980 nm and emission at 455 nm.

The detection threshold of the conventional malaria-nested PCR was determined using a ten-fold serial dilution series of *P. falciparum* NF54 culture samples ranging from 800 to 0.0.08 pg DNA per reaction (~3.4X10^4^ to ~3 parasites/reaction). The amplicons generated were detected by agarose gel electrophoresis and lateral flow UCP detection (Figure 2B). Figure 2A provides a schematic of the binding events occurring at the respective Test and Control lines for lateral flow detection. Negative controls lacked reactivity.

**Figure 3:**
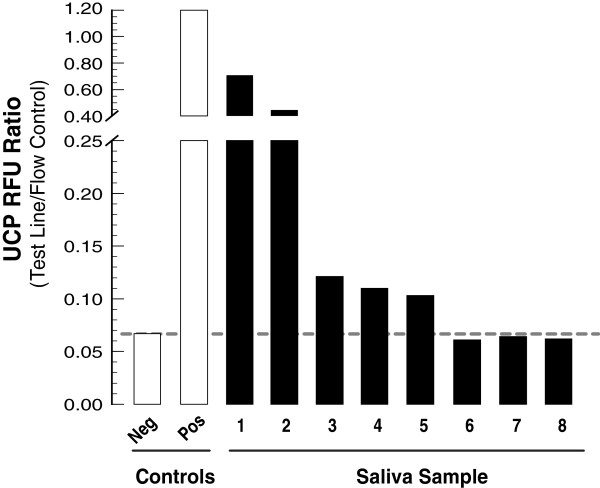
**UCP detection analysis of clinical malaria saliva samples using the semi-nested asymmetric PCR protocol.** UCP lateral flow assay: UCP reporter was used to detect the biotin-digoxigenin labelled amplicons generated using the single-step PCR amplification protocol. Parasite concentrations were determined using thick film microscopy of subject blood. The threshold is indicated as a horizontal dashed line.

**Figure 4:**
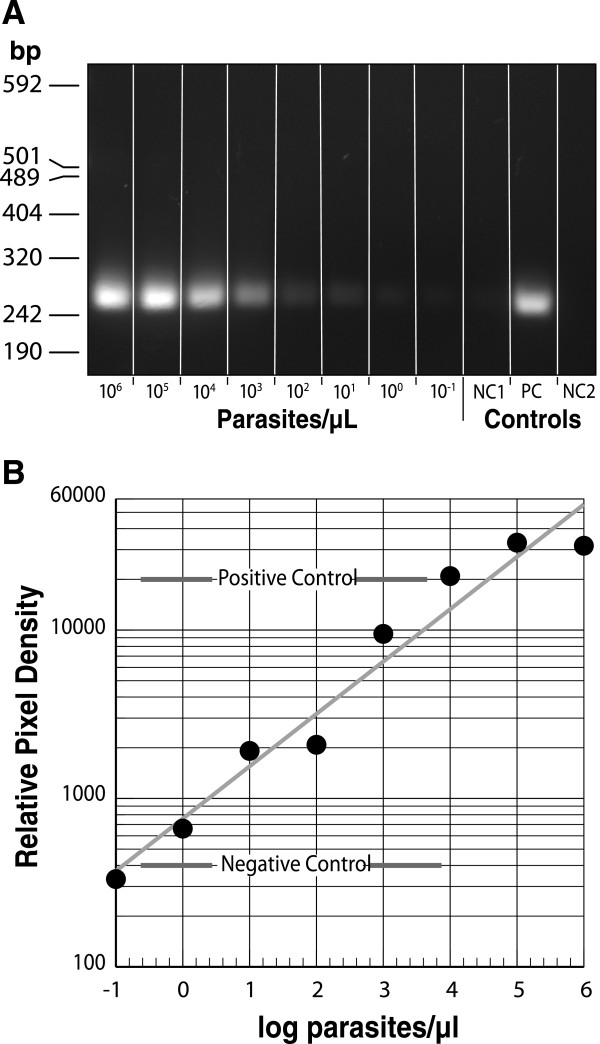
**LOD of parasite DNA using semi-nested PCR from six 3-mm diameter stored dried blood spots. A**) Amplicons are visualized in a 1.5% agarose gel stained with ethidum bromide. NC1 refers to a blood sample that was not spiked with any parasite and went through an identical DNA isolation and amplification to the rest the experimental samples. NC2 refers to PCR master mix to which PCR grade water was substitutes for eluted DNA as a control for contaminants in PCR reagents. **B**. Graph of the relative pixel density of the amplicon as a function of the parasite concentration.

DNA was extracted from saliva specimens (1 ml) obtained from negative and positive malaria patients with low-grade parasitaemia (ranging from microscopy negative to 3 × 10^3^ parasites/μl of peripheral blood), eluted in 200 μl, and 8 μl of the eluted DNA template amplified by the semi-nested PCR. The results obtained by gels and UCP LF assay of the clinical samples were concordant with the subject’s known malaria status and level of parasitaemia (Figure 3). These experiments successfully demonstrate proof-of-principle for uninterrupted single-tube PCR for detection of plasmodium DNA with both *P. falciparum*-positive laboratory control and clinical samples. In addition it is possible to utilize the semi-nested PCR approach for sensitive detection of *P. falciparum* in dried blood spots (Figure 4)*.*

The basis for the single-tube amplification is that the first nine annealing cycles favour efficient amplification of the primary U1/U2 product, which subsequently serves as template for the secondary U1/U4 amplicon, both during the primary and secondary reactions. The 252 bp amplicon is most efficiently amplified at the annealing temperature of the subsequent 25 cycles. The concentration of U1 primer is highest, as it is used both in the primary and secondary reactions; U2 has the lowest concentration for the initial short (nine) primary annealing cycles, which minimizes competition with the secondary primer U4; and U4 has a standard primer concentration to serve in the 25 secondary cycles. In this approach, the primary amplicon rate of production gets a head start, but then slows as it provides template for the second amplification.

## Discussion

The current experiments provide proof of principle for a malaria diagnostic that combines a quick semi-nested asymmetric PCR combined with a UCP lateral flow assay for the detection of *P. faciparum* DNA in saliva samples. A semi-nested asymmetric PCR that can detect *P. falciparum* DNA with a lower limit of detection comparable to that of the conventional nested PCR with both *P. falciparum* standard DNA as well as with saliva samples (Figure 3) was demonstrated. The sensitivity threshold for the original nested PCR strategy was equivalent to ~1-10 parasite/μl of peripheral blood sample [29,36], which surpasses routine microscopy (40 parasites/μl) and RDTs (100 parasites/μl) (Figure 4). The more specific gene-directed primers were chosen to pursue the semi-nested PCR. The first step in the semi-nested PCR is at high annealing temperature, which is more specific, while the second step is at a lower temperature and less specific. PCR performed at low specific conditions (low Ta) would generate non-specific products and/or false positives. The developed semi-nested asymmetric PCR minimizes the risk for contamination since it does not require sample carry-over to a second tube before starting the amplification of the nested fragment. Moreover, the semi-nested PCR only takes ~1.5 hours, which is ~40% of the total time taken to run conventional nested PCR [10,11]. Furthermore, in comparison to gel electrophoresis, LF allows a more convenient quantitative analysis suited for point-of-care applications. With a limited number of clinical saliva samples the feasibility of the assay was demonstrated to correctly identify positive samples. Additional studies are required to conclude whether this assay is a good point-of-care alternative for testing saliva-based samples rather than the conventional nested PCR developed for analysing blood-based samples in a dedicated diagnostic laboratory. The development of automated microfluidic devices capable of performing the above-described analysis of either a saliva or blood sample is in progress [37]. Recently a self-heating microfluidic chip for genotyping mosquitoes has been described by Liu *et al.*[38].

## Conclusions

This study demonstrates a semi-nested asymmetric PCR in a compressed time frame to detect *P. falciparum* DNA. The resulting digoxigenin-biotin tagged DNA amplicons are quantified using lateral flow and the sensitive UCP reporter technology. The assay reduces the chance of cross-contamination and can be readily configured as a POC assay using fresh or dried blood or saliva. The total assay time (including analysis) is reduced as compared to the conventional nested PCR approach with gel analysis. Integration of the new test into a microfluidic format using oral fluid has the potential to be used as an efficient non-invasive tool for POC applications, overcoming the limitations of microscopy and RDTs in detecting low-grade reservoirs of infection.

## Competing interests

The authors declare that they have no competing interests.

## Authors’ contributions

SYO, SM, EG, and CAB conducted experiments and analysed the results with WRA, PC, and DM, SYO, SM, PC, and WRA wrote the main paper. All authors discussed the results and implications and commented on the manuscript. All authors read and approved the final manuscript.
